# Effect of infliximab on mRNA expression profiles in synovial tissue of rheumatoid arthritis patients

**DOI:** 10.1186/ar2090

**Published:** 2006-11-29

**Authors:** Johan Lindberg, Erik af Klint, Anca Irinel Catrina, Peter Nilsson, Lars Klareskog, Ann-Kristin Ulfgren, Joakim Lundeberg

**Affiliations:** 1 School of Biotechnology, Department of Gene Technology, AlbaNova University Center, Royal Institute of Technology, Stockholm, Sweden; 2 Rheumatology Unit, Department of Medicine, Karolinska Institute, Karolinska University Hospital, Solna, Stockholm, Sweden

## Abstract

We examined the gene expression profiles in arthroscopic biopsies retrieved from 10 rheumatoid arthritis patients before and after anti-TNF treatment with infliximab to investigate whether such profiles can be used to predict responses to the therapy, and to study effects of the therapy on the profiles. Responses to treatment were assessed using European League Against Rheumatism response criteria. Three patients were found to be good responders, five patients to be moderate responders and two patients to be nonresponders. The TNF-α status of the biopsies from each of the patients before treatment was also investigated immunohistochemically, and it was detected in biopsies from four of the patients, including all three of the good responders. The gene expression data demonstrate that all patients had unique gene expression signatures, with low intrapatient variability between biopsies. The data also revealed significant differences between the good responding and nonresponding patients (279 differentially expressed genes were detected, with a false discovery rate < 0.025). Among the identified genes we found that MMP-3 was significantly upregulated in good responders (log_2 _fold change, 2.95) compared with nonresponders, providing further support for the potential of MMP-3 as a marker for good responses to therapy. An even more extensive list of 685 significantly differentially expressed genes was found between patients in whom TNF-α was found and nonresponders, indicating that TNF-α could be an important biomarker for successful infliximab treatment. Significant differences were also observed between biopsies taken before and after anti-TNF treatment, including 115 differentially expressed genes in the good responding group. Interestingly, the effect was even stronger in the group in which TNF-α was immunohistochemically detected before therapy. Here, 1,058 genes were differentially expressed, including many that were novel in this context (for example, CXCL3 and CXCL14). Subsequent Gene Ontology analysis revealed that several 'themes' were significantly over-represented that are known to be affected by anti-TNF treatment in inflammatory tissue; for example, immune response (GO:0006955), cell communication (GO:0007154), signal transduction (GO:0007165) and chemotaxis (GO:0006935). No genes reached statistical significance in the moderately responding or nonresponding groups. In conclusion, this pilot study suggests that further investigation is warranted on the usefulness of gene expression profiling of synovial tissue to predict and monitor the outcome of rheumatoid arthritis therapies.

## Introduction

Rheumatoid arthritis (RA) is a chronic inflammatory disease currently defined by clinical criteria [1], but knowledge is scarce on the pathogenetic pathways involved, and the variations in this respect between different patients [2]. Since the main inflammatory processes occur in the inflamed synovial tissue, it is assumed that detailed analysis of molecular events in inflamed synovia may provide additional clues regarding the pathways involved and may identify new biomarkers for RA [3,4]. A series of new targeted therapies have been introduced in recent years for the treatment of RA, and several more are anticipated [5-10]. The clinical response to currently available therapies is quite variable between patients, and although several of these treatments have well-defined molecular targets, such as TNF-α, our knowledge of the molecules and pathways that are affected *in vivo *is still limited [11]. Improved knowledge of individual patterns in the joints of patients and the effects of targeted therapies would provide new opportunities to elucidate the mode of action and to facilitate the design of individualized treatment strategies.

Current knowledge about interindividual differences in molecular patterns in inflamed RA joints originates from immunomorphological studies, which have shown both considerable heterogeneity between different patients [12] and certain striking common features, such as an abundance of macrophages and macrophage-derived proinflammatory cytokines in inflamed joints of RA patients [13,14]. Few studies, however, have presented clear evidence that immunomorphologically recognized molecular patterns may reflect important pathogenetic differences between patients, or that such differences could be used to predict responses to therapy to specific drugs [12,15,16]. New high-throughput approaches are therefore needed that can analyze molecular patterns in many samples taken repeatedly during the course of a disease process, before and after specific therapies. A highly promising approach is microarray analysis, which allows the expression levels of thousands of genes to be monitored simultaneously and can therefore increase out understanding of the processes involved. Microarrays have previously been used to investigate various aspects of RA [2,4,17-22] and its potential uses within the field were recently reviewed by Jarvis [23].

Using a microarray approach we have previously demonstrated that gene expression varies less between biopsies sampled from one patient than between biopsies sampled from different patients [24]. These findings indicated that it would be meaningful to analyze synovial samples taken from individuals before and after a specific treatment, and to investigate possible correlations between changes in molecular patterns and the successfulness of the therapy. In the study presented here we used microarrays to investigate mRNA expression patterns in arthroscopically obtained synovial biopsies before and after 2 months of treatment with infliximab. Ten patients that were treated at 0 weeks, 2 weeks and 6 weeks were investigated. The clinical responses of the patients were assessed using the EULAR (European League Against Rheumatism) response criteria [25], and synovial tissue from each patient had previously been immunohistologically analyzed [16] for the presence of detectable levels of TNF-α before treatment (except for patient 10, see Materials and methods). Real-time PCR was used to verify the transcriptional data.

## Materials and methods

### Patients

Ten patients (eight females and two males, median age 54 years, range 25–69 years) meeting the American College of Rheumatology criteria for RA were recruited for the study (Table 1). All patients received infliximab infusions of 3 mg/kg at week 0 and again after 2 weeks and 6 weeks. Five of these patients were receiving prednisolone in doses lower than 7.5 mg/day, and all 10 patients were treated with methotrexate to a maximum of 20 mg/week. Patients were assessed for the overall activity of the disease using DAS28 (disease activity score including a 28-joint count) values before and after 12 weeks of treatment. EULAR response criteria were used to determine the patient responses to therapy after 12 weeks of treatment.

Synovial biopsies were obtained from all patients during arthroscopy before and after a median of 9 weeks of treatment (with one exception, patient 7, from whom the second biopsy was obtained during open surgery). The biopsies obtained were taken from the site of inflammation, which was classified as being either close to cartilage or not close to cartilage (defined as <1.5 cm or >1.5 cm away from cartilage, respectively). All biopsies were snap-frozen in precooled isopentane within 2 minutes to ensure high RNA quality. One biopsy was taken before and after treatment from patients 1, 5, 7, 9 and 10, while multiple biopsies were retrieved before and/or after treatment from patients 2, 3, 4, 6 and 8. Since only small amounts of RNA were available for patient 9, the RNA from this patient was only used in the second series of hybridizations (see below). The average biopsy weight was 22 mg. The ethical committee at the Karolinska Hospital approved all experiments on human cells and tissues. Informed consent was obtained from all study participants.

### Additional immunohistochemical data for patient 10

Synovial biopsies from patient 10 were stained and scored for the presence of TNF-α as described by Ernestam and colleagues [16]. Briefly, TNF monoclonal antibodies 1 and 11 were scored using a semiquantitative four-point scale as follows: 0 = no positive cells, 1 = 1–10 positive cells, 2 = 11–100 positive cells, and 3 => 100 positive cells. The score for patient 10 was 1 (Table 1).

### RNA extraction

RNA was successfully extracted from all biopsies essentially as previously described [24]. Briefly, RNA was extracted from the biopsies using steel-bead matrix tubes and a tabletop FastPrep homogenizer (Qbiogene, Irvie, California, USA). After homogenization, the tubes were centrifuged and the water phase from each sample was collected and transferred to a Qiashredder (Qiagen, Venlo, Netherlands) columns to ensure complete homogenization. The RNA in the samples was purified on RNeasy spin columns (Qiagen) and was treated with DNAse H. The extracted RNA was eluted in RNase-free water. For further details see the KTH microarray core facility webpage [26] ('Preparation of RNA from tissues' under 'Protocols'). The average biopsy weight used for RNA extraction was 22 mg and the average RNA yield was 387 ng/μl in a volume of 30 μl. RNA quality was assured using the RNA 6000 Nano LabChip kit of the Bioanalyzer system from Agilent Technologies (Santa Clara, California, USA).

### RNA amplification

The extracted RNA was amplified by T7-based *in vitro *transcription using an Arcturus (Arcturus, Mountain View, California, USA) RiboAmp RNA amplification kit following the manufacturer's instructions. This procedure generates amplified RNA that consists mainly of sequences 250–1,800 bp long. Between 300 ng and 1 μg total RNA was used in each RNA amplification, and the average yield was 469 ng/μl in 11 μl H_2_O.

### RNA reference

Universal Human Reference RNA from Stratagene (La Jolla, California, USA) was used as reference RNA, amplified in the same manner as the sample RNA. The reference RNA was pooled before it was used for hybridization.

### Labeling and cDNA synthesis

Amplified RNA was labeled as previously described [24]. Briefly, amplified RNA was labeled using random hexamer primers in a cDNA synthesis reaction using Superscript III (Invitrogen, Carlsbad, California, USA) and an amino allyl-dUTP + dNTP mix. The mixture was incubated at 46°C for 2 hours. All purification steps were performed using a MinElute Reaction Cleanup Kit (Qiagen). The eluate from each sample was mixed with a dried portion of either Cy3 or Cy5 monoreactive esters (Amersham-Biosciences, Chalfont St Giles, UK) and was incubated for 30 minutes in a dark container at room temperature. After spin-column purification the samples were eluted and were ready for hybridization. For further information about the preparation of NHS-ester fluorophores and indirect labeling of cDNA, see the KTH microarray core facility webpage [26] (SOP 001 and SOP 002 at 'Protocols').

### cDNA microarray

The cDNA arrays used in this study have been previously described [24]. Briefly, the clones on the array (spotted inhouse) originate from the first 310 96-well plates of a commercial clone collection containing 46,000 sequence-verified human cDNA clones (Research Genetics, now Invitrogen). According to UniGene mapping performed in September 2005, based on GenBank accession numbers (29,717 on the whole chip), 23,300 out of the 30,000 spots on the chip had UniGene IDs, and 13,426 of these were unique. For more information about the chip, see the KTH microarray core facility webpage [26].

### Hybridization

The hybridization procedure has been previously described [24]. Briefly, after prehybridization, washing and centrifugation, the two samples (one Cy5-labeled and one Cy3-labeled) were pooled and hybridized in a water bath at 42°C for 14–18 hours. After hybridization, the slides were washed, centrifuged dry and scanned. For further information about the hybridization, see the KTH microarray core facility webpage [26] (SOP 003 under 'Protocols').

### Scanning and image processing

An Agilent G2565BA scanner was used to scan the slides and acquire 50 MB TIFF images of them. The scanner resolution was set at 10 μm. GenePix 5.1.0.0 (Axon Instruments, Union City, California, USA) was used to extract the raw signals from the TIFF images and to assign each spot an ID. Spots defined as 'Not Found' by GenePix were flagged with a negative flag (-50) and were removed downstream in the analysis. Spots with a clearly abnormal morphology due to the presence of dust particles or other factors were manually flagged as bad (-100) and were also removed in downstream analysis. No further processing of the slides was performed in GenePix. The data are available at ArrayExpress, a public repository for microarray data (accession number E-TABM-104) [27].

### Experimental design

Two series of hybridizations were performed. For each hybridization either RNA from two different samples or from one sample and reference RNA were labeled using different fluorophores (cy3 or cy5) and were hybridized onto the same array. Each hybridization was carried out using a dye-swapped technical replicate, which is a replicate with the same amplified RNA but with reversed fluorophores hybridized onto a separate array. The average correlation between the log_2 _fold changes (*M *values) of technical replicates was 0.8. In the first series of hybridizations, biopsies taken before treatment were hybridized in a common reference design to enable comparisons between patients. In the second series of hybridizations, biopsies taken before and after treatment were hybridized on the same chip in a direct design. A direct design has higher sensitivity since it provides better approximations of standard errors in comparison with the more common reference design, but the latter reference design allows more flexibility in downstream analysis [28].

### Data analysis

Data were analyzed as previously described [24]. Briefly, the data were mainly analyzed with the help of software packages [26,29-31] available in the R language [32]. After importing the result files (gpr files) produced by GenePix into R language, unreliable spots with abnormal physical properties were removed using four filters (described in [24] – more information can be found at the KTH package webpage [26]). On average 3,826 spots were removed by the filters, leaving approximately 26,174 spots for downstream analysis. After filtering, the slides were normalized using print-tip Loess normalization [33].

A moderated *t *test implemented in the LIMMA (Linear Models for Microarray Data) package [29,34] was used to identify differentially expressed (DE) genes. The probability of falsely identifying DE genes due to problems arising from multiple testing was controlled using the false discovery rate approach [35], in which a *q *value (analogous to a *P *value) is assigned to each gene. A cutoff value was set at 0.025, which means that the expected proportion of false positives should be less than 0.025% among differentially expressed genes. When making comparisons instead of merely taking the average of replicates, the duplicateCorrelation function [36] available in LIMMA [29] was used to acquire an approximation of gene-wise variance. This retains valuable information about the variance when fitting a linear model to the data in order to identify differentially expressed genes.

Biopsies were taken both close to cartilage and not close to cartilage from patients 2, 3, 4 and 6. Data from these biopsies were not averaged. These data were instead treated as different biological replicates in the tests for differentially expressed genes since these two site types are known to represent different cellular populations [37,38]. Functions available in Bioconductor [39] were used, with the help of Gene Ontology (GO) [40] to find 'biological process' GO terms over-represented in sets of DE genes. Only GO terms represented by more than five genes in the set of DE genes investigated were used to reduce the amount of false positives. Each GO category is given a *P *value, essentially utilizing Fisher's exact test [41]. The *P *values were corrected for multiple testing using the false discovery rate approach [35]. Several hierarchical clusterings were performed [42], in which 1 – Pearson correlation was used as the distance measure (for more detailed information see [24]).

### Real-time PCR

Up to 1 μg total RNA was used in the first-strand synthesis reaction depending on the amount of material left, to which 2 μl 20TVN primer (4 μg/μl; Operon, Huntsville, Alabama, USA) was added to prime the reaction. The volume was adjusted to 15.5 μl using RNase-free water, was mixed and was then incubated for 10 minutes at 70°C to denature the RNA. The samples were then incubated for another 2 minutes on ice and were spun briefly. A cDNA synthesis mixture (12.5 μl) consisting of 6 μl of 5 × first-strand buffer, 3 μl of 0.1 M dithiothreitol, 2 μl Superscript III (Invitrogen) and 1.5 μl of 10 mM dNTP mix (Amersham) were added to each sample. The whole mixture was then incubated at 46°C for first-strand synthesis. After 1 hour the temperature was increased to 70°C for 15 minutes to terminate the reaction. Then 2 U RNase H (Invitrogen) was added to degrade the RNA. After RNAse treatment the temperature was increased to 70°C to inactivate the RNAse. All samples were then diluted with RNase-free water to a final volume of 200 μl.

Real-time PCR was performed using the iCycler system from Biorad (Hercules, California, USA). Each reaction was performed with 3.0 μl template, 12.5 μl iQSYBR Green Supermix (Biorad), 300 nM primer and water to adjust the final volume to 25 μl. The PCR amplification steps were applied in the following conditions: 3 minutes at 95°C, and then 40 cycles of 20 seconds at 94°C, 30 seconds at 60°C and 1 minute at 72°C. This was followed by melt curve analysis to ensure specific amplification. The signal was calculated in all experiments using the 'PCR base line subtracted RFU' and a cutoff value of 300 was used to establish threshold cycle values.

All primers were designed using Primer Express software (PE Applied Biosystems, Foster City, California, USA) and amplicons were designed to span exon-exon junctions to minimize contamination by genomic DNA. Primer sequence information is available in Additional file 1. Differences in relative expression levels between samples were calculated using the ΔΔCt method [43] and all samples were analyzed in triplicate. β-Actin was used as a reference gene. The following genes (with GenBank IDs indicating the sequences present on the microarray) were assayed in the real-time PCR: *CCL19 *(GenBank AA680186), *CCL3 *(GenBank AA677522), *CXCL10 *(GenBank AA878880), *CXCL9 *(GenBank AA131406), *CXCL1 *(GenBank W42723), *CXCL3 *(GenBank AA935273), *IL1RN *(GenBank T72877), *FCGR1A *(GenBank AA453258), *CXCL1 *(GenBank W42723), *CXCL14 *(GenBank AI016051) and *TNF*-α (GenBank AA699697).

## Results

Two series of hybridizations were performed to investigate the differences between patients before treatment and to determine possible correlations between gene expression patterns that could be used to predict clinical responses, and to investigate gene expression patterns before and after treatment to identify potential molecular explanations for variations in the clinical responses. To facilitate these two tasks we determined and analyzed genes that were DE in different comparisons obtained from the microarray experiments. As already described in Materials and methods, a gene was considered DE if *q *< 0.025. EULAR response criteria were used to assess the clinical responses.

### Hierarchical clustering of biopsies before treatment

**Figure 1:**
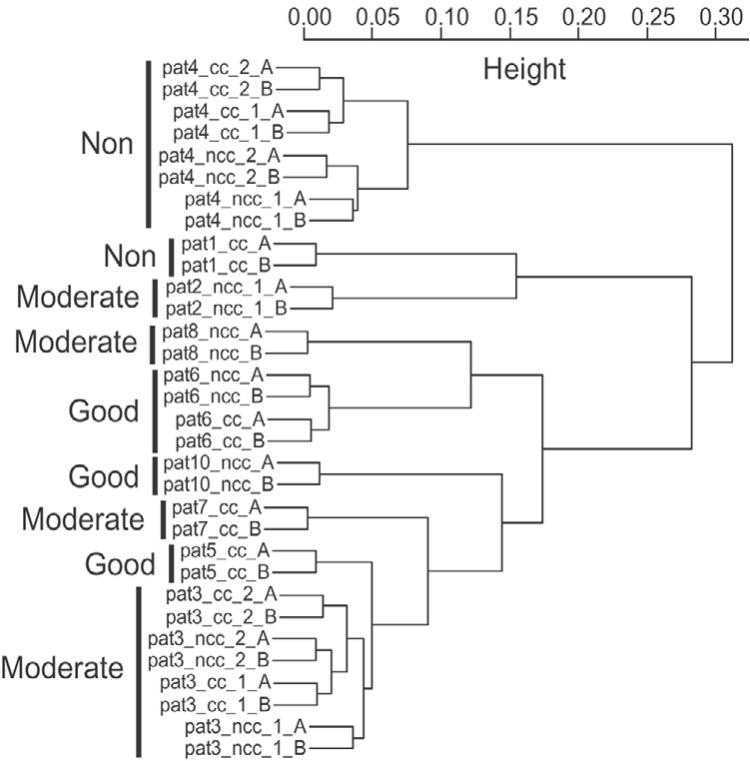
Hierarchical cluster dendrogram of the biopsies taken before treatment. A hierarchical cluster dendrogram of the 876 genes with *M *values (log_2 _fold change) with interquartile range > 1 in the first series of hybridizations. The clinical response of each patient is given in the plot (European League Against Rheumatism response criteria). pat, patient; cc, close to cartilage; ncc, not close to cartilage; biopsy 1 or 2 for each biopsy type, technical replicate A or B.

Before comparing patients with differing clinical responses, a hierarchical clustering was performed on the 876 genes that had *M *values (sample versus universal RNA reference) with interquartile ranges > 1 (Figure 1) in the first series of hybridizations. This clustering was performed to obtain an overview of the differences between the technical replicates (that is, replicates of the same amplified RNA aliquot with reversed fluorophores, denoted A and B), the resampled biopsies (denoted 1 or 2) and the biological replicates in the form of different biopsy types (denoted close to cartilage or not close to cartilage) from the patients. The data show that all patients had a unique gene expression signature with low patient intravariability between biopsies, in accordance with previous work [24], but the clustering did not separate the patients clearly into groups correlating with their EULAR responses.

### Differential gene expression between different responder groups

**Figure 2:**
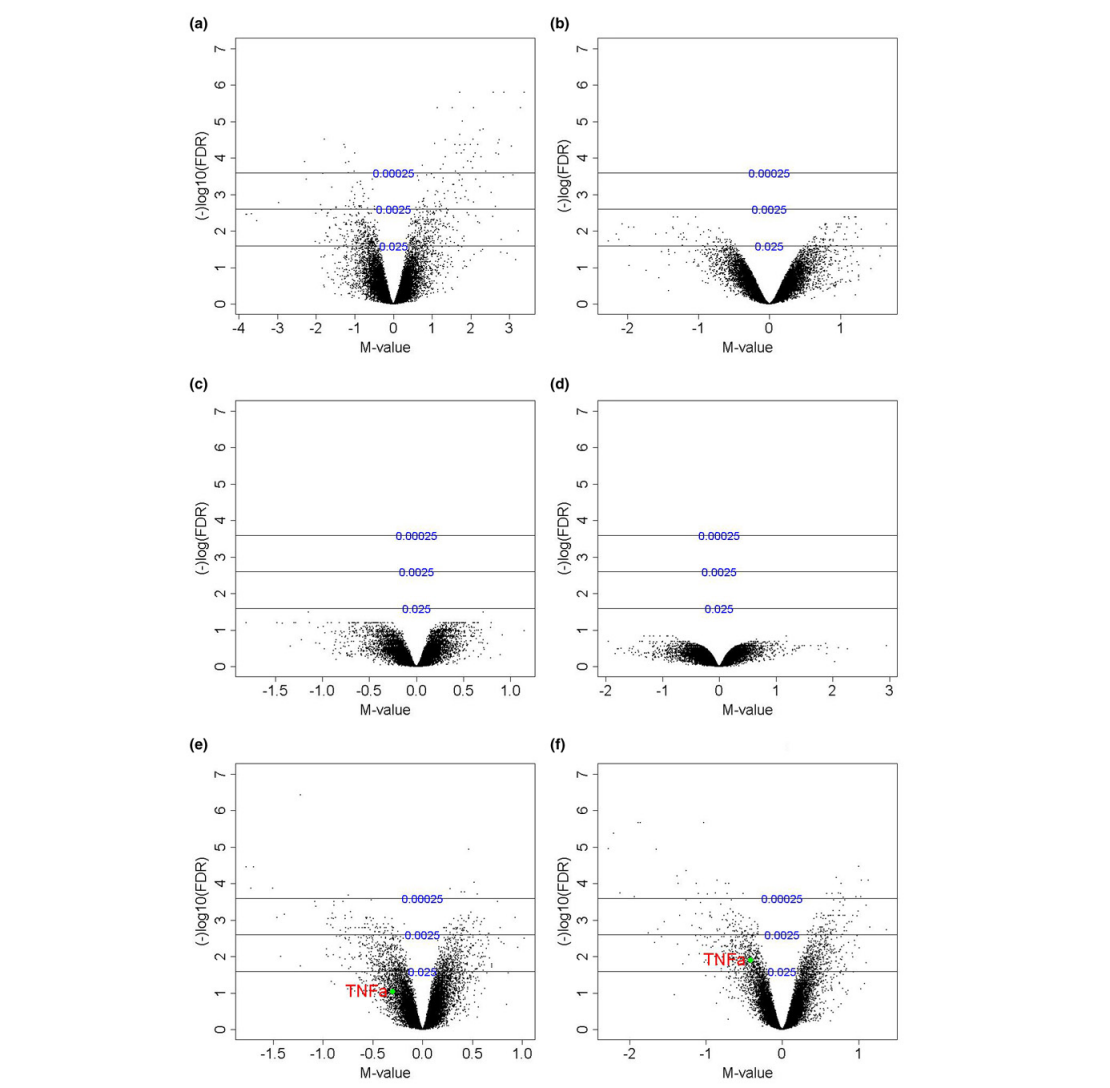
Volcano plots displaying differential expression. The *y *axis corresponds to the negative false discovery rate on a log_10 _scale. The *x *axis displays the *M *value (log_2 _fold change). Differential expression (proportion of false positives < 0.025): **(a) **between TNF-positive patients and nonresponders (685 differentially expressed (DE) genes) before treatment, **(b) **before and after treatment in the good responders (115 DE genes), **(c) **before and after treatment in the moderate responders (0 DE genes), **(d) **due to treatment in the nonresponders (no DE genes), **(e) **before and after treatment in all patients (974 DE genes), and **(f) **before and after treatment in the TNF-positive group of patients (1058 DE genes). TNF-α is highlighted in (e) and (f). FDR, false discovery rate.

To further investigate whether the differential gene expression data retrieved from the biopsies taken before treatment correlated with the clinical responses, series of comparisons were performed between good responders (patients 5, 6 and 10), moderate responders (patients 2, 3, 7, 8 and 9) and nonresponders (patients 1 and 4). A total of 279 DE genes were obtained when good responders were compared with nonresponders. No genes reached statistical significance if good responders were compared with moderate responders and nonresponders. In contrast, 382 DE genes were statistically significant when the nonresponders were compared with those that displayed a response (moderate or good). Twelve genes were found to be DE between the patients in whom TNF-α was detected before treatment immunohistochemically (patients 3, 5, 6 and 10, hereafter denoted TNF-positive patients) and the TNF-negative patients. Interestingly, the highest number of DE genes (685) was found in the comparison of TNF-positive patients and nonresponders (Figure 2a). These results indicate that there were similarities within the defined responder groups and clear distinctions between the pretreatment gene signatures of good responders and nonresponders (Table 2).

### Effect of treatment with infliximab

The effect of treatment with infliximab was investigated in the second series of hybridizations, in which RNA from biopsies taken before and after treatment was hybridized in a direct design. The results are presented in Figure 2b–f. These plots display the distribution of genes obtained with different statistical thresholds (*y *axis, negative false discovery rate on a log_10 _scale) in correlation to their *M *value (change due to treatment). In the good responders 115 genes were DE (Figure 2b), whereas no genes reached significance in the moderate responders or the nonresponding patients (Figure 2c,d). A total of 974 genes were DE (Figure 2e) when testing for the effect of treatment using all patients, but the largest effect (1,058 DE genes) was observed in the TNF-positive group of patients (Figure 2f, Additional file 8).

### Over-representation analysis

**Figure 3:**
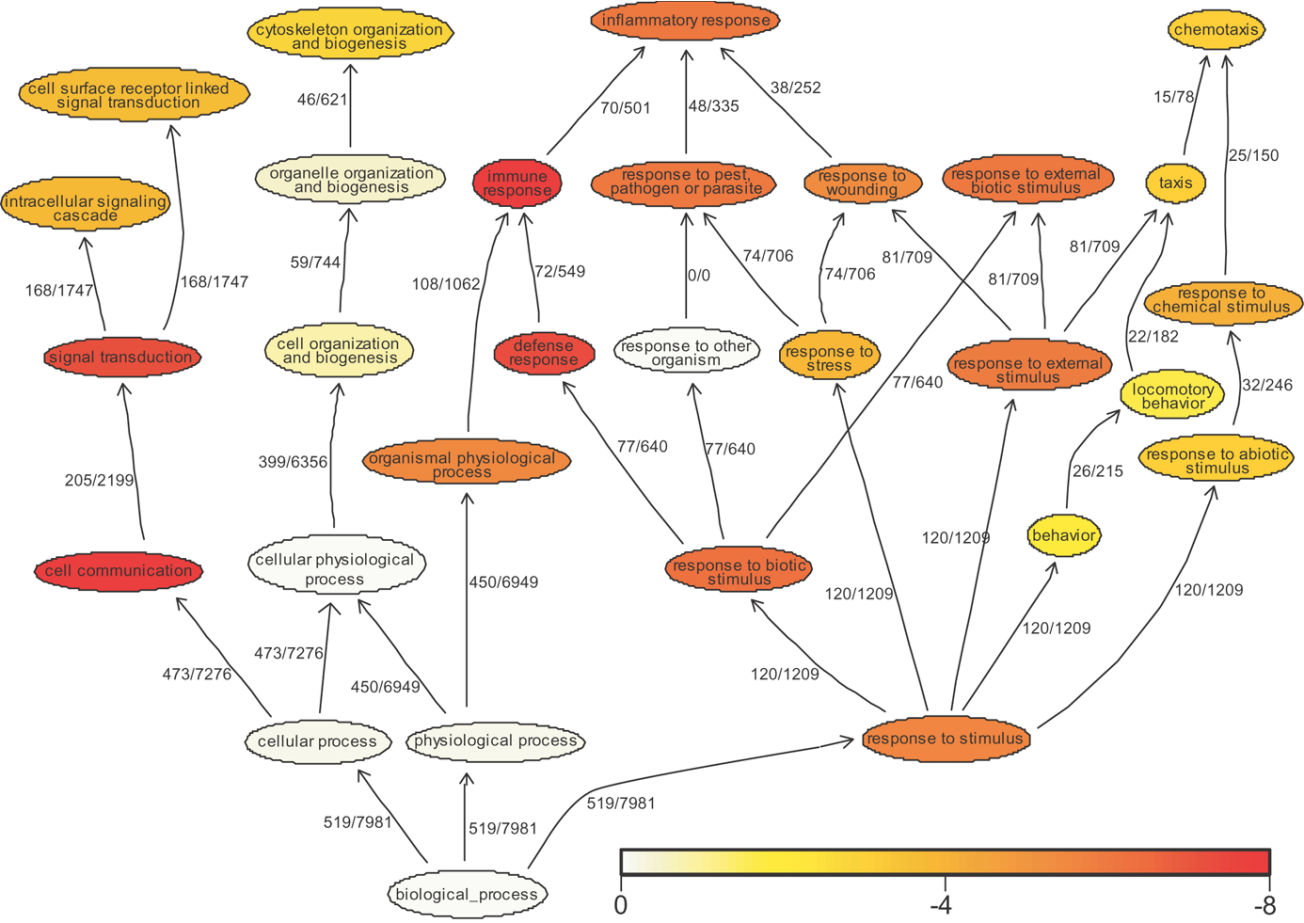
Gene Ontology analysis of the genes differentially expressed before and after treatment in the TNF-positive group of patients. The 20 most significant biological processes (Gene Ontology categories) and their parent terms are shown. The color of each node illustrates significance and can be interpreted in the scale bar, which displays the false discovery rate on a log_10 _scale. The arrows indicate the parent to child direction. The numbers of each arrow demonstrate the amount of unique Entrez Gene IDs [57] among the DE genes that are mapped to the parent term, and the number of unique Entrez Gene IDs among all genes present on the chip that are mapped to the parent term.

To investigate whether any biological processes were over-represented among the genes that were DE before and after treatment in the TNF-positive patients and good responders, GO analysis was performed as described in Materials and methods [39]. For the TNF-positive patients (Figure 3) this revealed significant themes related to inflammatory disease and relevant for anti-TNF treatment, such as immune response (GO:0006955), cell communication (GO:0007154), signal transduction (GO:0007165) and chemotaxis (GO:0006935). Similar themes were also significant for good responders (see Additional files 9, 10  and 11).

### Verification

**Figure 4:**
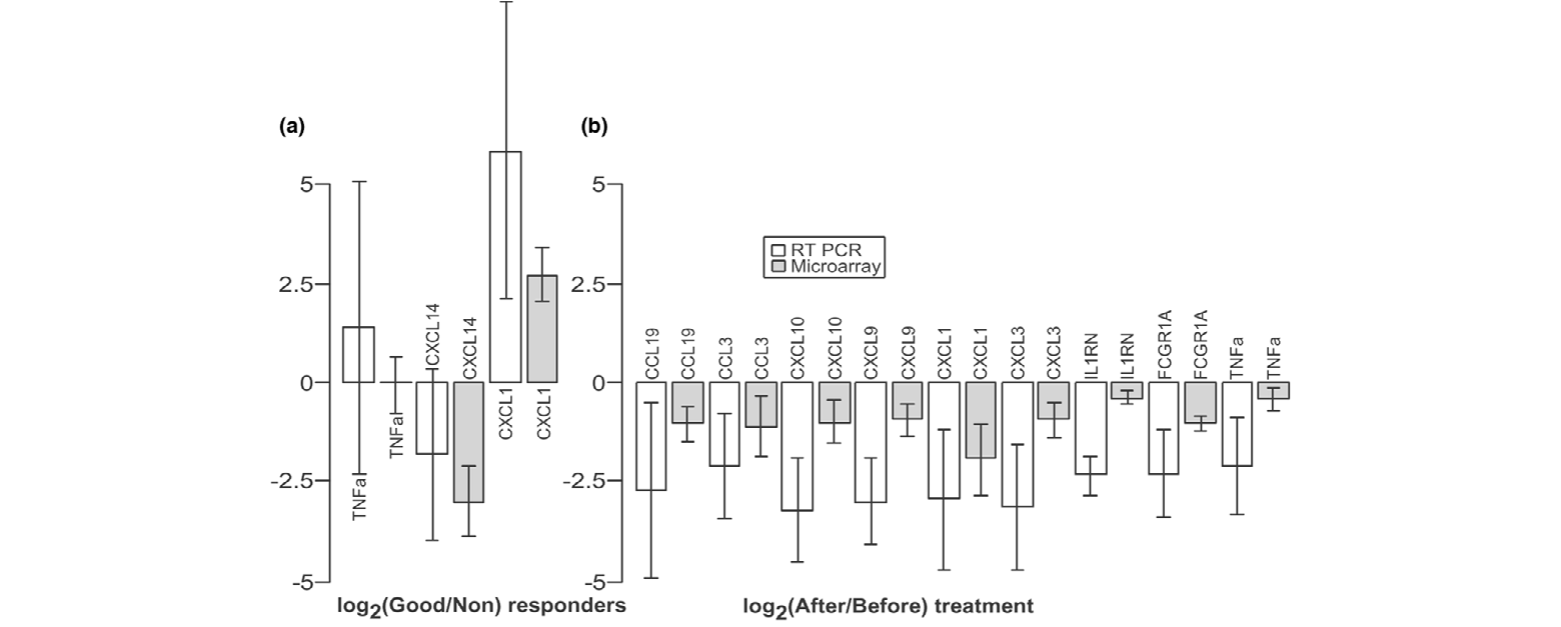
Real-time PCR results. **(a) **Real-time results for four genes in the first series of hybridizations comparing TNF-positive patients and nonresponders. **(b) **Real-time results for nine genes in the second series of hybridizations in the TNF-positive patients. The whiskers display the standard deviations in both (a) and (b).

Real-time PCR was performed on unamplified total RNA. Three DE genes were verified before treatment between the TNF-positive patients and the nonresponders. As shown in Figure 4a, TNF-α was not DE according to results obtained using the microarray platform. The real-time PCR results indicated that TNF-α was upregulated in the patients in whom TNF-α was detectable before treatment, but the variation was high. The results from both approaches agreed in the direction of changes in CXCL14 and CXCL1 levels. The change of CXCL14 was higher according to results obtained with the microarray platform, but this is probably due to the high variation in the real-time PCR data for this gene. Verification experiments using real-time PCR were also performed on nine genes that were DE before and after treatment in TNF-positive patients. As displayed in Figure 4, the microarray and real-time PCR results agreed on the expression direction (either up-regulated or downregulated) of all genes but, as shown previously [44,45], the changes were underestimated by the microarray relative to the real-time PCR results.

## Discussion

This investigation was a pilot study of the feasibility of using gene expression profiling to predict responses to TNF-blocking and to assess the effects of TNF-blocking on gene expression patterns in synovial tissues of RA patients. The results reveal significant differences between different response groups before treatment and identify genes that were significantly affected by treatment in patients who responded well to therapy and in patients where TNF could be detected immunohistochemically before treatment.

The first objective of this study was to investigate whether the gene expression signatures of the arthroscopic biopsies taken before treatment could predict the outcome of the treatment. In the hierarchical cluster (Figure 1) based on the gene expression signature before treatment, the patients with moderate/good EULAR responses tend to group together. A possible explanation for the limited overall degree of separation may be that the selection of the subset of genes chosen for clustering was based on variations between all biopsies, instead of being based on response groups where it was not clear *a priori *if the gene expression signature related to the TNF response would be relatively strong or weak. We therefore evaluated another strategy – investigating genes that are differentially expressed in groups of patients, classified on the basis of their response to therapy according to EULAR response criteria. Clustering of DE genes between, for example, nonresponders and good/moderate responders resulted in a clear distinction in the dendrogram between the two groups (see Additional file 2).

We also investigated differences in gene expression between the groups in which TNF was present and those in which TNF was absent in the inflamed joint tissue immunohistochemically. The obvious assumption was that TNF-dependent pathways can be assumed to play a more active role in the inflammation of patients when this molecule is present in substantial amounts prior to therapy. Previous research has suggested that the number of TNF-α-producing cells detected immunohistochemically in the rheumatoid synovium may indicate the response to infliximab therapy [12,16]. In nonresponding patients, other molecules, and thus different pathogenic pathways, might be sustaining the chronic inflammation. A particularly large difference in genes expressed before and after therapy was seen in those patients who showed both TNF expression before therapy and a good clinical response, providing indirect additional support for this hypothesis. Analysis of differentially expressed genes before and after treatment in all patients simultaneously resulted almost as many significant genes were detected (974 DE genes) as in the TNF-positive group, but as exemplified by TNF-α in figures 2e and 2f important observations can be missed if confounding cohorts used to test for a specific effect with unaffected/moderately effected patients. Thus, even though one would be able to detect differences due to treatment using all patients, the heterogeneity of the group will make it difficult to differentiate small differences from noise (TNF-α, *q *= 0.09 using all patients). Another obvious conclusion is that other targets need to be identified for the treatment of nonresponders.

Several interesting GO terms were over-represented in the genes differentially expressed before and after treatment in the TNF-positive group (Figure 3). The genes mapped to significant GO terms are available in Additional file 10. Previous work has documented several mechanisms of action of TNF antagonists in RA, the most prominent being downregulation of synovial inflammation [12,46], reduction of cell migration into the synovium [47], modulation of cartilage and bone destruction markers [48-50] and induction of apoptosis [51]. It is relevant to note in this context that our GO analysis identified modulation of chemotaxis, immune functions, inflammatory responses and signal transduction as potentially relevant mechanisms. Our findings therefore confirm the relevance of the previously described mechanisms of action for this type of RA therapy. Moreover, these findings may facilitate the selection of better target molecules for further studies regarding the mechanisms of action of TNF antagonists (such as the FcgRs, Toll-like receptors and the IL18 receptor). This study might also aid in suggesting new pathways to be studied in a more focused approach with confirmation at the protein levels and investigation of the clinical significance.

Many of the chemokines that were downregulated due to treatment have previously been detected in the synovial membrane and analyzed by immunohistochemical techniques. Some of them are novel in this context, however; for example, CXCL3, which was down-regulated following treatment, and CXCL14, which had a higher level of expression in nonresponders before treatment. The downregulation of CXCL3, a chemoattractant of neutrophils, natural killer cells and cytotoxic T cells [52], is consistent with earlier studies showing that the influx of inflammatory cells is downregulated by TNF therapy [53]. CXCL14 has not been reported to be significant in the context of rheumatic tissue but has been shown by Shurin and coworkers [54] to be a potent chemoattractant and activator of dendritic cells, which have been proposed to play a role in the initiation and perpetuation of RA by presentation of arthritigenic antigens to autoreactive T cells [55]. CXCL1, known to be an angiogenic protein, is particularly interesting since its expression was higher in good responders than in nonresponders before treatment, and was also found to be downregulated in good responders following treatment. CXCL1 has previously been shown [56] to be induced by TNF-α and IL-1β. In this study CXCL1 was found to be downregulated, as was TNF-α. IL-1β was also downregulated (*q *value = 0.026), but did not reach our threshold for statistical significance (*q *value = 0.025).

Another interesting finding is related to the expression of MMP-3 at the synovium level. We have previously shown that high levels of MMP-3 in serum before treatment may indicate good responses to therapy, as evaluated by the change in DAS28 (disease activity score including a 28-joint count) values [48]. Our analysis of differential expression between good and poor responders revealed an upregulation of MMP-3 in the synovial tissue of good responders relative to nonresponders. These findings suggest that further confirmatory studies on larger numbers of patients are warranted, with the ultimate aim of identifying a biological marker that could be used to predict responses to treatment.

## Conclusion

The present paper describes the use of an mRNA expression array technique to analyze synovial biopsies arthroscopically obtained before and after treatment with infliximab. Three major results were obtained. First, we confirmed our previous observations that intrapatient variations in biopsies are smaller than interpatient variations, reflecting unique mRNA signatures of each patient rather than tissue heterogeneity. Secondly, we demonstrated significant pretreatment differences in gene expression between patient groups, the largest being between the patients with positive immunohistochemical scores for TNF-α and nonresponding patients. Thirdly, we also observed genes differentially expressed before and after infliximab therapy among the good responders, but the largest differences were found when testing for effect of treatment in the patients where TNF-α could be detected before treatment.

In summary, these results show the potential of using gene expression profiling to elucidate the effects of anti-TNF treatment in RA patients using synovial biopsies and indicate the possibility of identifying gene expression signatures predictive of good responses.

## Abbreviations

bp = base pair; DE = differentially expressed; EULAR = European League Against Rheumatism; GO = Gene Ontology; IL = interleukin; LIMMA = Linear Models for Microarray Data; *M *value = log_2 _fold change; PCR = polymerase chain reaction; RA = rheumatoid arthritis; TNF = tumor necrosis factor.

## Competing interests

The authors declare that they have no competing interests.

## Authors' contributions

JL performed parts of the microarray-related laboratory work, contributed to the data analysis and wrote parts of the article. EaK performed the arthroscopic surgery and contributed to the data analysis. AIC participated in data analysis and writing of the article. PN was responsible for chip production. LK was involved in planning the project, data analysis and writing of the article. A-KU contributed to the planning and design of the project and participated in both the data analysis and writing of the article. JL was involved in project design, analysis of laboratory results, data analysis and writing of the article.

## Supplementary Material

Additional file 1 An Excel file containing the sequence of primers used in the real-time PCR. Click here for file

Additional file 8 An Excel file containing a list of the DE genes due to treatment in the TNF-positive group of patients. Click here for file

Additional file 9 A pdf file containing an image of GO analysis of the DE genes due to treatment in EULAR good responders. Click here for file

Additional file 10 An Excel file containing a list of GO categories (biological process) of DE genes due to treatment in TNF-positive patients. Each category is assigned a *q *value to establish over-representation of the specific category among the DE genes. Click here for file

Additional file 11 An Excel file containing a list of GO categories (biological process) of DE genes due to treatment in EULAR good responders. Each category is assigned a *q *value to establish over-representation of the specific category among the DE genes. Click here for file

Additional file 2 A pdf file containing figures for Table 2. Click here for file

Additional file 3 An Excel file containing a list of DE genes of EULAR good responders versus nonresponders for Table 2. Click here for file

Additional file 4 An Excel file containing DE genes of TNF-positive patients versus TNF-negative patients for Table 2. Click here for file

Additional file 5 An Excel file containing DE genes of EULAR nonresponders versus responders for Table 2. Click here for file

Additional file 6 An Excel file containing DE genes of TNF-positive patients versus EULAR nonresponders for Table 2. Click here for file

Additional file 7 An Excel file containing GO analysis of DE genes between TNF-positive patients and nonresponders for Table 2. Click here for file

## Figures and Tables

**Table 1:** Clinical data of patients

Patient	Diagnosis/rheumatoid factor	Sex, age (years)	Time of diagnosis	Duration of arthritis	DMARD	Corticosteroids	EULAR response criteria	TNF staining before treatment^a^
1	RA, positive	Female, 55	18 years	NA	Methotrexate 7.5 mg/week	Prednisolone 5 mg/day	None	0
2	RA, positive	Female, 52	6 years	2 weeks	Methotrexate 17.5 mg/week	Prednisolone 5 mg/day	Moderate	0
3	RA, positive	Female, 58	2 years	6 months	Methotrexate 7.5 mg/week	Prednisolone 7.5 mg/day	Moderate	1
4	RA, negative	Female, 57	4 years	2 weeks	Methotrexate 10 mg/week	None	None	0
5	RA, positive	Male, 56	9 years	1 month	Methotrexate 7.5 mg/week	Prednisolone 5 mg/day	Good	1
6	RA, negative	Female, 69	1 year	1 year	Methotrexate 15 mg/week	Prednisolon 5 mg/day	Good	1
7	RA, positive	Female, 66	14 years	1 week	Methotrexate 10 mg/week	None	Moderate	0
8	RA, positive	Female, 66	10 years	3 weeks	Methotrexate 12.5 mg/week	None	Moderate	0
9	RA, positive	Male, 25	7 months	3 days	Methotrexate 17.5 mg/day	Prednisolon 20-7.5 mg/day	Moderate	0
10	RA, positive	Female, 35	26 years	1 year	Methotrexate 17.5 mg/day	Prednisolon 7.5 mg/day	Good	1

DMARD, disease-modifying antirheumatic drug; EULAR, European League Against Rheumatism; NA, not available; RA, rheumatoid arthritis.

^a^The scoring system is described in Materials and methods, Additional immunohistochemical data for patient 10.

**Table 2:** Results of different comparisons between responder groups using biopsies retrieved before treatment

Comparison	Differentially expressed genes
Good responders versus nonresponders	279^a^
Good responders versus moderate responders/nonresponders	0
Nonresponders versus good responders/moderate responders	382^b^
TNF-positive patients versus TNF-negative patients	12^a^
TNF-positive patients versus nonresponders	685^c^

^a^Volcano plot and a list of the differentially expressed genes is available in Additional files 2, 3 and 4.

^b^Volcano plot, hierarchical cluster and a list of the differentially expressed genes is available in Additional files 2 and 5.

^c^A list of the differentially expressed genes and Gene Ontology analysis of the differentially expressed genes is available in Additional files 6 and 7.
