# Supplementary figures and images for: Effect of infliximab on mRNA expression profiles in synovial tissue of rheumatoid arthritis patients
Source: Arthritis Res Ther. 2006 Nov 29;8(6):R179. doi: 10.1186/ar2090 (PMC1794525; doi:10.1186/ar2090)

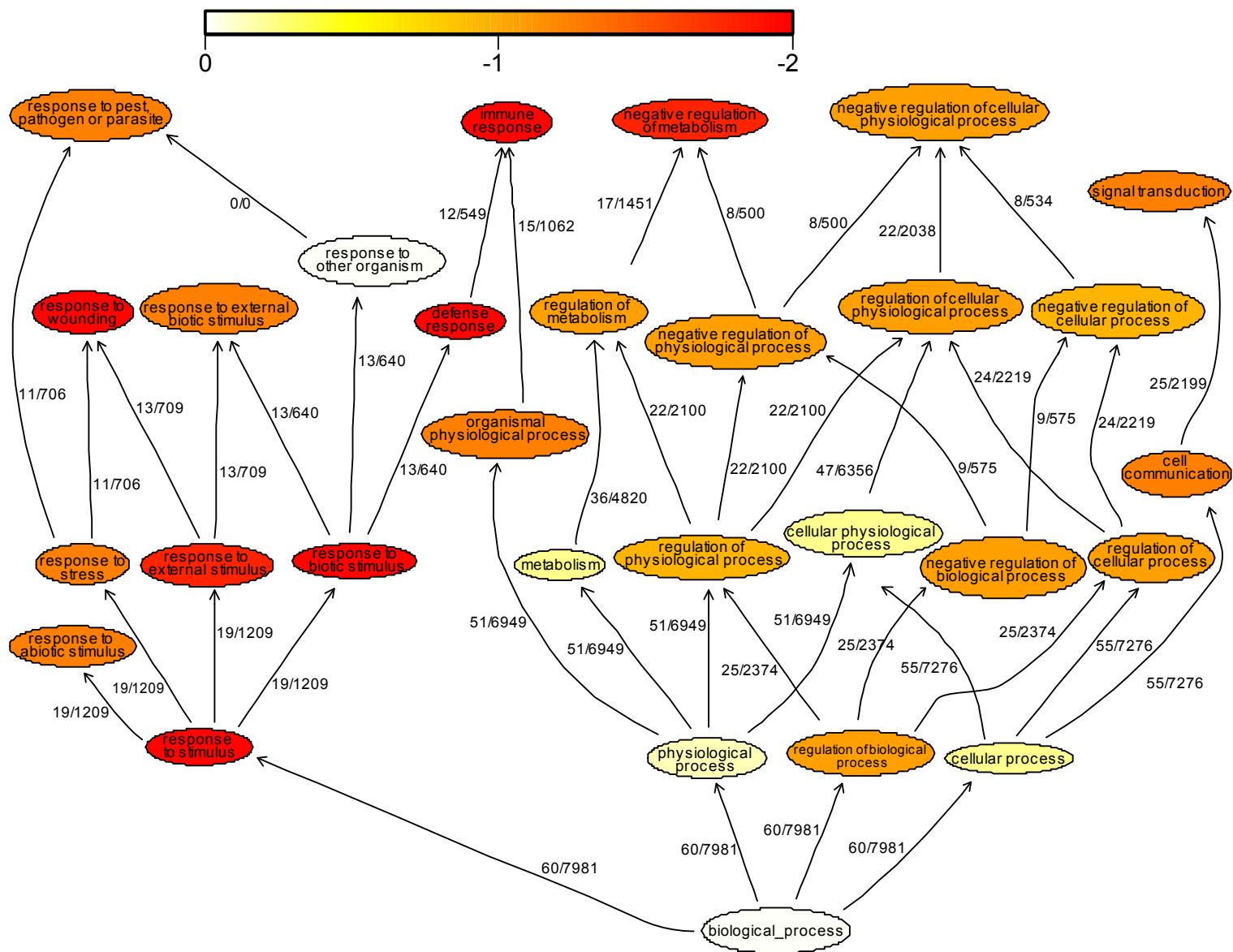

Supplement: Additional file 9 — A pdf file containing an image of GO analysis of the DE genes due to treatment in EULAR good responders. [file ar2090-S9.pdf]
