# Supplementary material for: Effect of infliximab on mRNA expression profiles in synovial tissue of rheumatoid arthritis patients
Source: Arthritis Res Ther. 2006 Nov 29;8(6):R179. doi: 10.1186/ar2090 (PMC1794525; doi:10.1186/ar2090)
Supplement: Additional file 2 — A pdf file containing figures for Table 2. [file ar2090-S2.pdf]

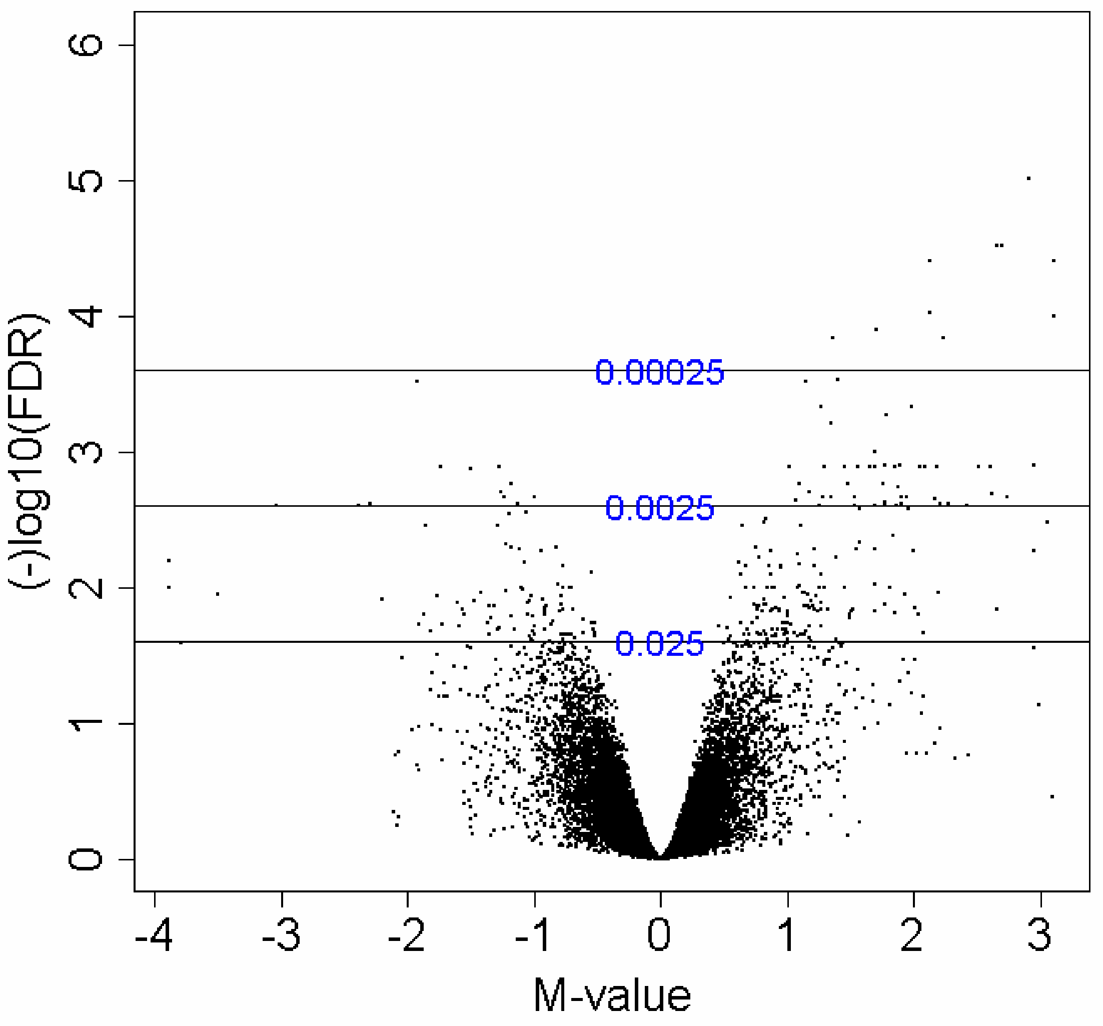

Volcano plot displaying differential expression between EULAR good responders and EULAR non-responders. The Y-axis corresponds to the negative false discovery rate on a $\log_{10}$ scale. The X-axis displays the M-value representing the $\log_2$(fold change). 279 genes were DE (proportion of false positives < 0.025).

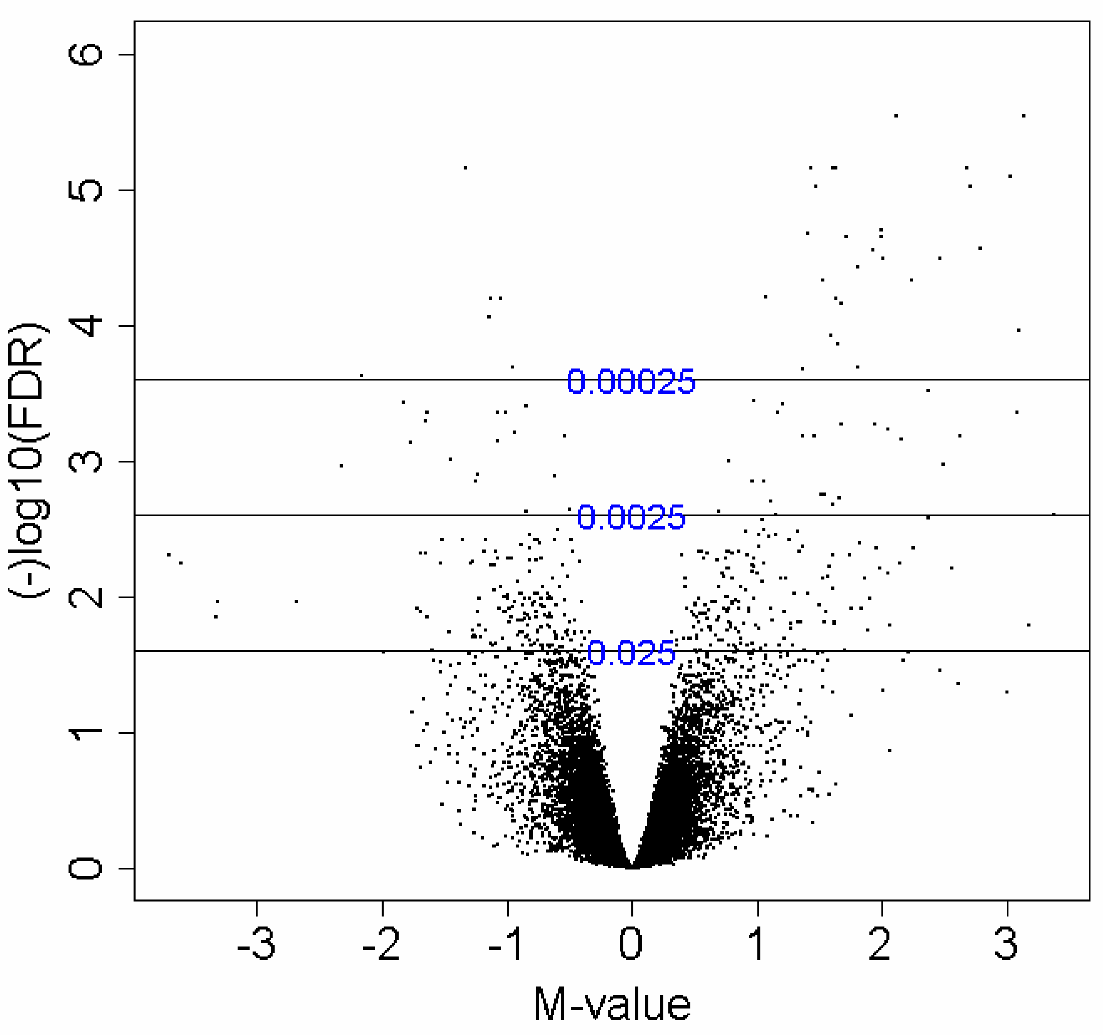

Volcano plot displaying differential expression between EULAR non-responders and EULAR good/moderate responders. The Y-axis corresponds to the negative false discovery rate on a $\log_{10}$ scale. The X-axis displays the M-value representing the $\log_2$(fold change). 382 genes were DE (proportion of false positives < 0.025).

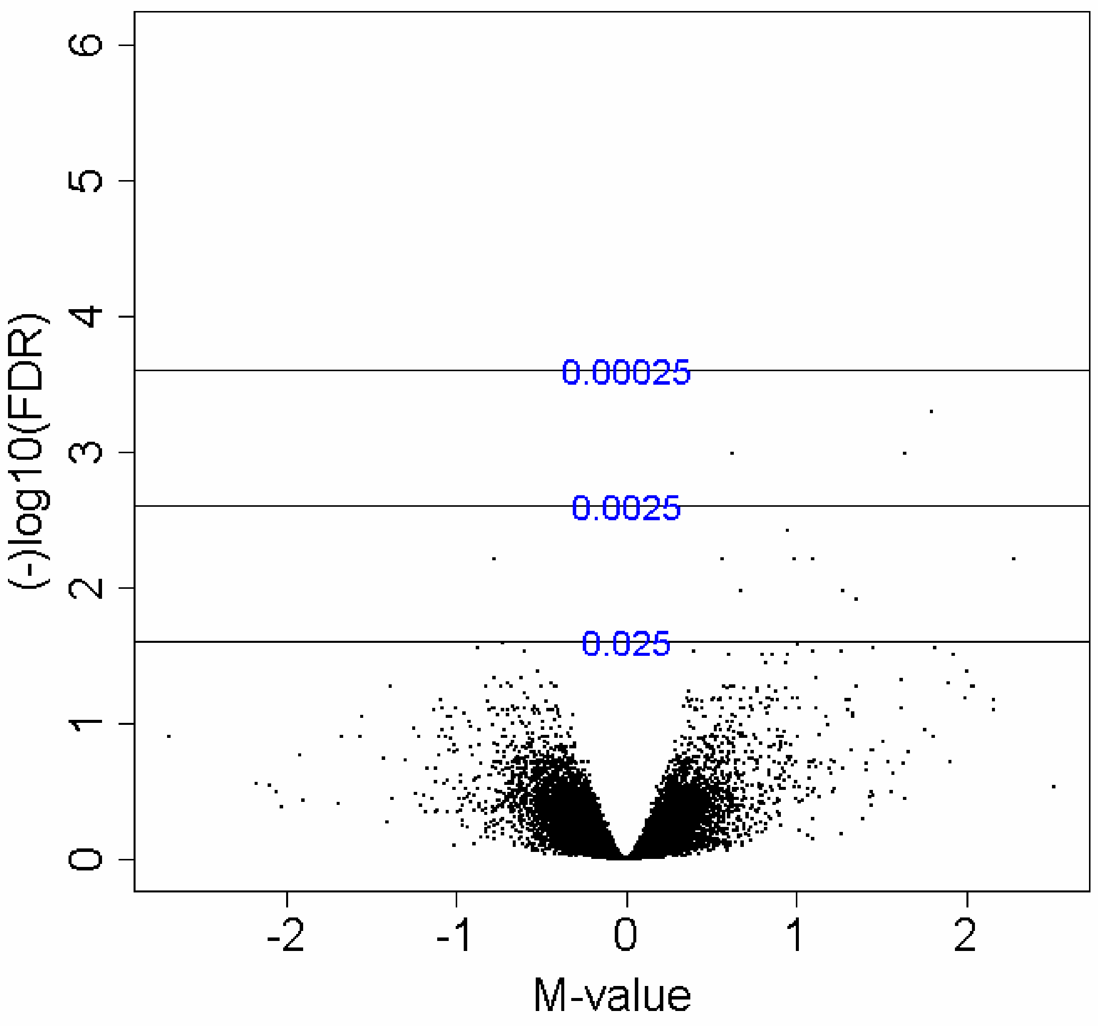

Volcano plot displaying differential expression between the TNF-positive and TNF-negative patients. The Y-axis corresponds to the negative false discovery rate on a $\log_{10}$ scale. The X-axis displays the M-value representing the $\log_2$(fold change). 12 genes were DE (proportion of false positives < 0.025).

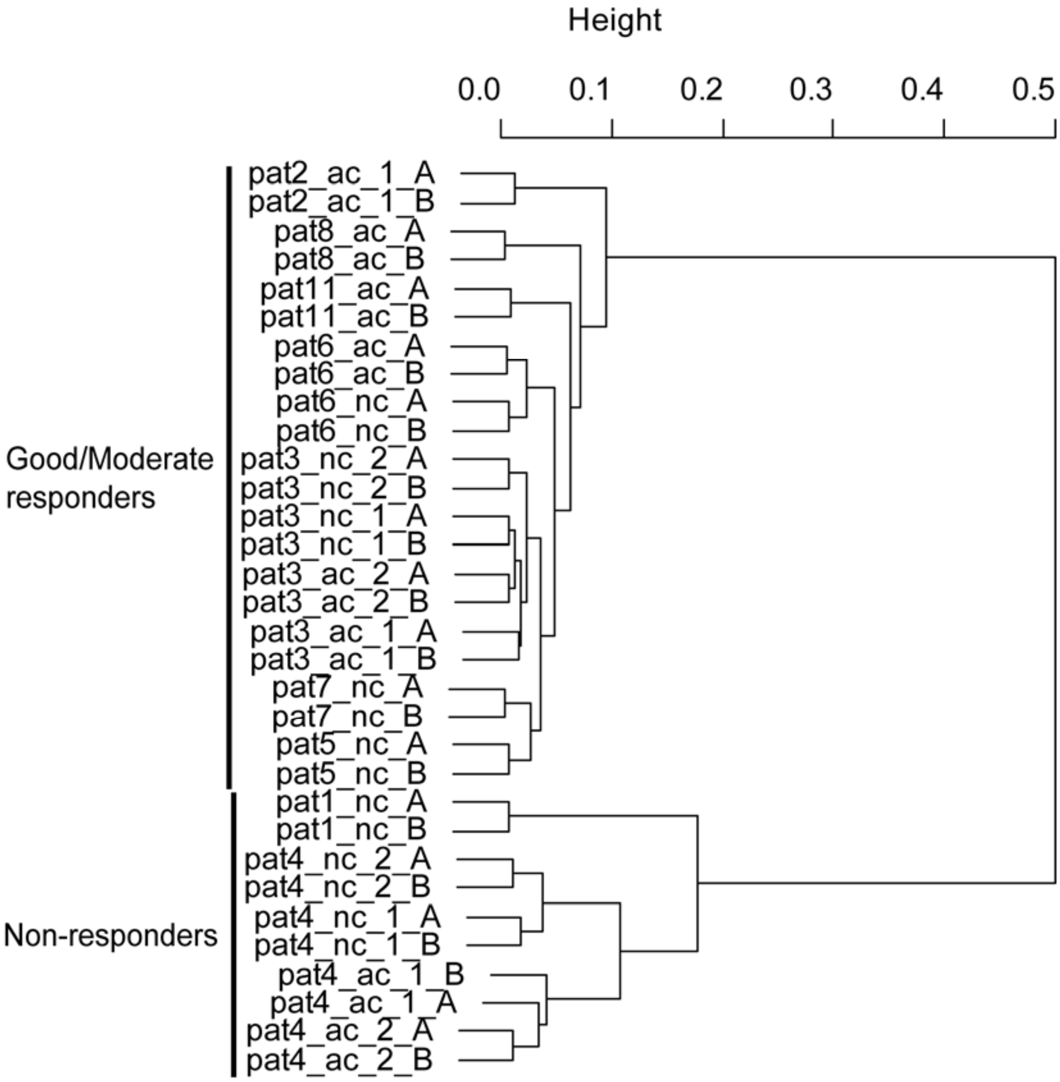

Hierarchical cluster of the patients before treatment using the genes differentially expressed between EULAR non-responders and EULAR Good/Moderate responders. Abbreviations, pat – patient, cc – close to cartilage, ncc – not close to cartilage.
